# NFκB signaling drives pro-granulocytic astroglial responses to neuromyelitis optica patient IgG

**DOI:** 10.1186/s12974-015-0403-8

**Published:** 2015-09-30

**Authors:** Margaret E. Walker-Caulfield, Yong Guo, Renee K. Johnson, Christina B. McCarthy, Patrick D. Fitz-Gibbon, Claudia F. Lucchinetti, Charles L. Howe

**Affiliations:** Department of Neurology, Mayo Clinic, 200 First St. SW, Rochester, MN 55905 USA; Department of Health Sciences Research, Mayo Clinic, Rochester, MN USA; Department of Neuroscience, Mayo Clinic, Rochester, MN USA; Department of Immunology, Mayo Clinic, Rochester, MN USA

**Keywords:** PR-957, Bortezomib, NMO, Reactive astrocyte, NFκB

## Abstract

**Background:**

Astrocytes expressing the aquaporin-4 water channel are a primary target of pathogenic, disease-specific immunoglobulins (IgG) found in patients with neuromyelitis optica (NMO). Immunopathological analyses of active NMO lesions highlight a unique inflammatory phenotype marked by infiltration of granulocytes. Previous studies characterized this granulocytic infiltrate as a response to vasculocentric complement activation and localized tissue destruction. In contrast, we observe that granulocytic infiltration in NMO lesions occurs independently of complement-mediated tissue destruction or active demyelination. These immunopathological findings led to the hypothesis that NMO IgG stimulates astrocyte signaling that is responsible for granulocytic recruitment in NMO.

**Methods:**

Histopathology was performed on archival formalin-fixed paraffin-embedded autopsy-derived CNS tissue from 23 patients clinically and pathologically diagnosed with NMO or NMO spectrum disorder. Primary murine astroglial cultures were stimulated with IgG isolated from NMO patients or control IgG from healthy donors. Transcriptional responses were assessed by microarray, and translational responses were measured by ELISA. Signaling through the NFκB pathway was measured by western blotting and immunostaining.

**Results:**

Stimulation of primary murine astroglial cultures with NMO IgG elicited a reactive and inflammatory transcriptional response that involved signaling through the canonical NFκB pathway. This signaling resulted in the release of pro-granulocytic chemokines and was inhibited by the clinically relevant proteasome inhibitors bortezomib and PR-957.

**Conclusions:**

We propose that the astrocytic NFκB-dependent inflammatory response to stimulation by NMO IgG represents one of the earliest events in NMO pathogenesis, providing a target for therapeutic intervention upstream of irreversible cell death and tissue damage.

**Electronic supplementary material:**

The online version of this article (doi:10.1186/s12974-015-0403-8) contains supplementary material, which is available to authorized users.

## Background

Neuromyelitis optica (NMO) is a severe, generally relapsing disease of the central nervous system (CNS) characterized by optic neuritis and transverse myelitis with longitudinally extensive spinal cord lesions [1, 2]. The identification of an NMO-specific autoantibody (NMO IgG) and aquaporin 4 (AQP4) as an antigenic target of this antibody defined NMO as a distinct disease with unique pathogenic and pathological characteristics [3]. AQP4, the principle water channel in the CNS, is densely expressed on perivascular astrocytic endfeet and is crucial for bidirectional water transport and normal CNS homeostasis [4, 5]. The AQP4 expression pattern and distribution of NMO-specific lesions [6] suggest that astrocytes are a cellular target of NMO IgG and that NMO is a primary astrocytopathy [7].

Immunopathological analyses of active NMO lesions define a unique vasculocentric pattern of complement activation and granulocytic infiltration involving both eosinophils and neutrophils [8, 9]. Characteristic IgG deposition and complement activation on the adluminal surface of the vasculature corresponds to the location of the astrocyte endfeet that envelop the blood vessels [8]. Evidence from ex vivo and in vitro studies is currently interpreted in support of a model for NMO pathogenesis wherein NMO IgG gains entry into the CNS, binds to AQP4 on astrocytic foot processes, and induces complement activation and deposition of the terminal membrane attack complex, resulting in astrocyte injury and death that leads to recruitment of eosinophils and neutrophils into the lesions [4, 10]. In this model, complement-mediated astrocyte death is the key driver of chemokine, cytokine, and toxic effector production in lesions that results in the recruitment of macrophages that then induce demyelination and the death of oligodendrocytes and neurons [11]. This model defines granulocytic recruitment as a *consequence* of complement-mediated astrocyte death. However, recent evidence from human tissue indicates that many NMO lesions are non-destructive but highly inflammatory, with prominent activation of parenchymal microglia and perivascular macrophages, infiltration of neutrophils, and degranulation of infiltrated eosinophils in the absence of astrocyte death, terminal complement deposition, or overt tissue destruction [9, 12]. This suggests that alternative mechanisms may be responsible for granulocytic recruitment in early NMO lesions.

Astrocytes are central mediators of general CNS homeostasis, participating in and controlling key metabolic cascades that are vital for normal neuronal function. Astrocytes are also active participants in the pathogenesis of numerous CNS diseases, modulating local inflammatory responses, controlling blood–brain barrier function, and serving as a source of chemokines and cytokines [13, 14]. Such astrocyte-initiated inflammatory responses set the stage for leukocyte-mediated feedback loops that elicit profound neuropathology during infection, inflammation, autoimmunity, and trauma. Recently, we observed that stimulation of primary rat astrocyte cultures with serum or IgG isolated from NMO patients resulted in the release of the potent pro-granulocytic chemokine CCL5, with essentially no release stimulated by serum from MS or systemic lupus erythematosus (SLE) patients [7]. These data suggest that astrocytes respond directly to NMO patient-derived IgG, and that the stimulated chemokine response is disease-specific and pro-granulocytic. Based on these observations, we hypothesize that the astrocytic inflammatory response to stimulation by NMO IgG represents one of the earliest pathogenic events in NMO, preceding severe and irreversible cell death and tissue damage.

## Methods

### Histopathology analysis

Histopathology was performed on archival formalin-fixed paraffin-embedded autopsy-derived CNS tissue from 23 patients clinically and pathologically diagnosed with NMO or NMO spectrum disorder. Five-micrometer-thick sections were stained with hematoxylin and eosin (H&E), luxol fast blue, and periodic acid–Schiff or Bielschowsky silver impregnation. Immunohistochemistry was performed using primary antibodies against proteolipid protein (PLP) (1:500, Serotec), glial fibrillary acidic protein (GFAP) (1:100, Dako), and AQP4 (1:250, Sigma). C9neo was detected using monoclonal clone B7 (1:200) or polyclonal anti-C9neo (1:200), both a gift of Prof. Paul Morgan, Cardiff, UK.

A topographical map was made in order to define regions of interest based on the following: (1) stage of demyelinating activity (active demyelination, inactive demyelination, remyelination, periplaque white matter, or normal appearing white matter); (2) the extent of tissue damage, graded as none, mild (tissue vacuolation with mild microglial reaction), moderate (damaged and disorganized parenchymal cell components with obvious macrophage infiltration), or marked (prominent parenchymal cell loss or cystic lesions); (3) the nature of the astrocytic reaction based on GFAP staining and hypertrophy of astrocytic processes or the presence of dystrophic astrocytes [15]; (4) the presence or absence of complement deposition; and (5) the loss of AQP4 expression.

Eosinophils and neutrophils were identified based on morphological characteristics using H&E-stained sections. Eosinophil infiltration was measured semi-quantitatively in regions of interest and categorized as follows: mild = 1–3 cells per high power field (HPF) (40× objective lens); moderate = 4–10 cells/HPF; or marked >10 cells/HPF. Neutrophil infiltration was categorized as follows: mild = 1–3 cells/HPF; moderate = 4–20 cells/HPF; or marked >20/HPF.

All features of interest were captured as categorical data. Each feature was summarized in a contingency table and cross-classified according to the semi-quantitative assessment of granulocyte infiltration. To test for associations, the contingency tables were analyzed using log-linear regression models in the framework of generalized estimating equations that employed an “exchangeable” correlation structure in order to account for repeated observations among patients [16]. Each contingency table summarized region-level data such that an individual patient could contribute multiple regions to the data set. Intra-patient regional correlations were controlled for in the generalized estimating equations. All analyses were performed using R statistical software package version 3.0.2.

### Patient serum processing and IgG purification

Blood was drawn from patients or healthy volunteers and IgG was isolated from sterile-filtered, heat-inactivated serum samples as previously described [7]. For the present study, results were generated using purified IgG from five different pools prepared since 2011 (Additional file 1: Table S1). Representative results were generated from NMO patient sera pooled from 5 males and 36 females ranging in age from 14 to 79, with a median age of 48 (Additional file 2: Table S2). Control sera were pooled from age- and sex-matched donors (Additional file 3: Table S3). All treatments with human IgG were at 100 μg/mL [7].

### Mouse primary mixed glial cultures

Mixed glial cultures were prepared from P1-P3 Balb/c mouse pups, as described [17]. Cells were plated at 1.3 × 10^5^ cells/cm^2^ on poly-L-lysine hydrobromide. After 4 days in vitro, flasks were shaken to remove microglia and oligodendroglia. The astrocyte-enriched cultures were incubated for an additional 22 days and were then replated at 5.2 × 10^4^ cells/cm^2^ on poly-D-lysine. For all biochemical measurements, cells were stimulated starting at 31 days in vitro.

### Immunostaining and imaging

Cells were immunostained with mouse anti-GFAP antibody (Millipore, MAB360) at 1:200 and anti-NFκB p65 antibody (Cell signaling, 8242) at 1:400, as described [7]. Images were acquired using an LSM780 inverted confocal microscope (Carl Zeiss) and Zen software. Z-stacks were rendered into maximum intensity projections in ImageJ. All images were collected under identical conditions within a given experiment.

### Microarray

RNA samples were assessed by Agilent for integrity, purity, and concentration. Samples passing quality control were analyzed on Illumina mouse WG-6 v 2.0 expression BeadChips in the Mayo Clinic Medical Genome Facility Gene Expression Core. Expression data were analyzed using Excel and MatLab [7]. Heatmaps and hierarchical clusters were derived using Gitools v2.2.2 and pathway identification was performed using Ingenuity Pathway Analysis.

### Immunoblotting

Cells were serum-starved overnight prior to stimulation with NMO IgG or control IgG then lysed in RIPA buffer containing protease/phosphatase inhibitors. Cell lysates (10–30 μg) were run on 4–15 % Criterion Tris–HCl gels (Biorad). After transfer, blots were probed using anti-IκB-α (Cell Signaling 9242), anti-phosphorylated IκB-α (Cell Signaling 2895), anti-p65 (Cell Signaling 8242), anti-NUP98 (Cell Signaling 2598), or anti-tubulin (Sigma T9026) antibodies.

### ELISA

Following stimulation of cells, supernatants were collected, clarified, and stored as aliquots at −80 °C until analysis. Mouse CCL5, CCL2, CXCL1, and CXCL2 were detected in the supernatants using ELISA construction kits (Antigenix America).

### Statistics

α = 0.05 and β = 0.2 were established a priori. Post hoc power analysis was performed for all experiments and significance was only considered when power ≥0.8. Statistical analysis was performed using SigmaStat (Systat Software). Normality was determined by the Shapiro–Wilk test and normally distributed data were checked for equal variance. Parametric tests were only applied to data that were both normally distributed and of equal variance. The Student–Newman–Keuls pairwise comparison test was used for all post hoc sequential comparisons. The figures show representative results from at least two separate experiments performed in triplicate using independent cell cultures and purified IgG. Over the course of this study, five different NMO patient pools were utilized for the preparation of purified IgG (Additional file 1: Table S1). As shown in Additional file 4: Figure S1, similar results were attained using different patient serum pools.

### Study approval

All cell culture-based experiments were performed using materials approved by the Mayo Clinic institutional animal care and use committee. All studies were conducted in accordance with the United States Public Health Service’s Policy on Humane Care and Use of Laboratory Animals. The Mayo Clinic institutional review board approved the use of human materials. All subjects provided written informed voluntary consent after the nature and possible consequences of the study were explained.

## Results

### Granulocytic infiltration in NMO pathology is not dependent on terminal complement complex formation, active demyelination, or tissue destruction

**Fig. 1:**
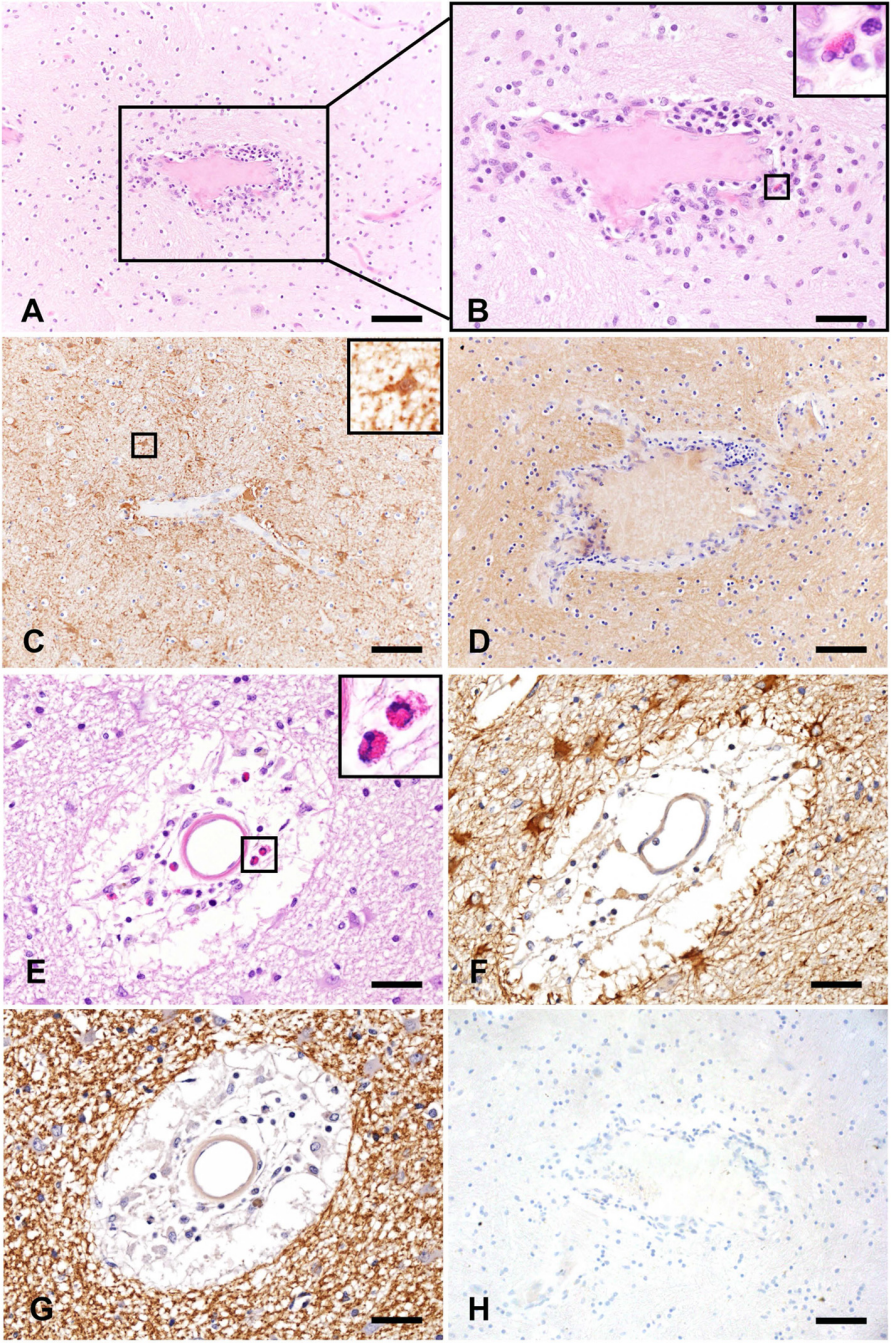
Granulocytic infiltrate occurs in the absence of demyelination, terminal complement complex formation, and overt tissue destruction in NMO white matter. **a** H&E staining reveals robust perivascular inflammation in the white matter of autopsy tissue collected from an NMO patient (scale bar = 100 μM). **b** An enlarged view of (**a**) and the *magnified inset* confirm the presence of eosinophils as a component of this perivascular infiltration (scale bar = 50 μM). **c** Staining for the astrocyte marker GFAP demonstrates the presence of reactive astrocytes with abnormal morphology in close association with granulocytic perivascular infiltration in a section consecutive to **a**. The *inset* highlights the increased size of a GFAP+ astrocyte (scale bar = 100 μM). **d** The perivascular granulocytic infiltration and astrocyte reactivity are present in a non-demyelinating NMO lesion as shown by the presence of intact proteolipid protein (PLP) staining in a section consecutive to **a** and **c** (scale bar = 100 μM). **e** A second example of perivascular granulocytic infiltration involving neutrophils and eosinophils (*inset*) in the white matter of an NMO patient is shown by H&E staining. The *inset* highlights the presence of eosinophils (scale bar = 50 μM). **f** Staining for the astrocyte marker GFAP in a section consecutive to **e** confirms the presence of numerous reactive astrocytes proximal to the perivascular inflammation (scale bar = 50 μM). **g** PLP staining reveals that in a section consecutive to (**e**) and (**f**) the myelin is intact, indicating that granulocytic infiltrate is found in the absence of demyelination (scale bar = 50 μM). **h** The complete absence of staining for the terminal complement protein C9neo in a section consecutive to **e**, **f**, and **g** shows that granulocytic recruitment to this site is not dependent on formation of the terminal complement complex (scale bar = 100 μM)

To determine whether granulocytic infiltration in NMO is dependent on formation of the terminal complement complex, demyelination, or tissue destruction, as the conventional model of NMO pathogenesis suggests, we semi-quantitatively assessed the extent of eosinophil and neutrophil infiltration in 1048 regions in 337 blocks from 23 NMO patients. In a subset of lesions, we observed granulocyte infiltration (Fig. 1a, b, e) in the absence of terminal complement complex formation, demyelinating activity, and tissue destruction (Fig. 1). C9neo served as a marker for terminal complement deposition, and although granulocyte infiltration was statistically associated with the presence of complement deposition (*p* = 0.012), 31 % of regions with mild infiltration, 20 % of regions with moderate infiltration, and 4 % of regions with marked infiltration were negative for C9neo (Fig. 1h). With regard to demyelinating activity, we found a statistically significant association between active demyelination and granulocytic infiltration (*p* < 0.001). However, the presence of granulocytes was not entirely dependent on demyelinating activity, as we observed mild, moderate, or marked infiltration in 9 % of NMO lesions devoid of demyelination (Fig. 1d, g). Finally, in regions with mild, moderate, or marked granulocyte infiltration, there was no statistically significant association between the extent of tissue damage and infiltration (*p* = 0.99) (Fig. 1), suggesting that granulocytic trafficking is not strictly in response to the presence or extent of tissue damage in NMO. These observations support the hypothesis that recruitment of neutrophils and eosinophils into the CNS may be one of the earliest consequences of NMO IgG binding to the surface of astrocytes. Furthermore, we observed profound astrocyte reactivity in proximity to infiltrating neutrophils and eosinophils (Fig. 1c, f), supporting our hypothesis that early astrocytic responses to NMO IgG may drive granulocytic recruitment and infiltration that precedes irreversible astrocytic death, demyelination, and tissue damage.

### Stimulation with NMO IgG elicits an inflammatory and pro-granulocytic transcriptional response in astroglial cultures

Considering our observations of early granulocyte recruitment in NMO (Fig. 1), we specifically asked what constitutes the glial response to stimulation with NMO IgG and whether this response is associated with granulocytic recruitment. Previously, we reported the robust induction of an immunological response in rat astrocyte cultures to stimulation with both NMO patient serum and isolated IgG [7]. Here, we utilized a mouse glial culture system to expand our understanding of the cellular responses to NMO IgG and to provide a platform for the identification of potentially targetable inflammatory signaling pathways that drive such responses.

**Fig. 2:**
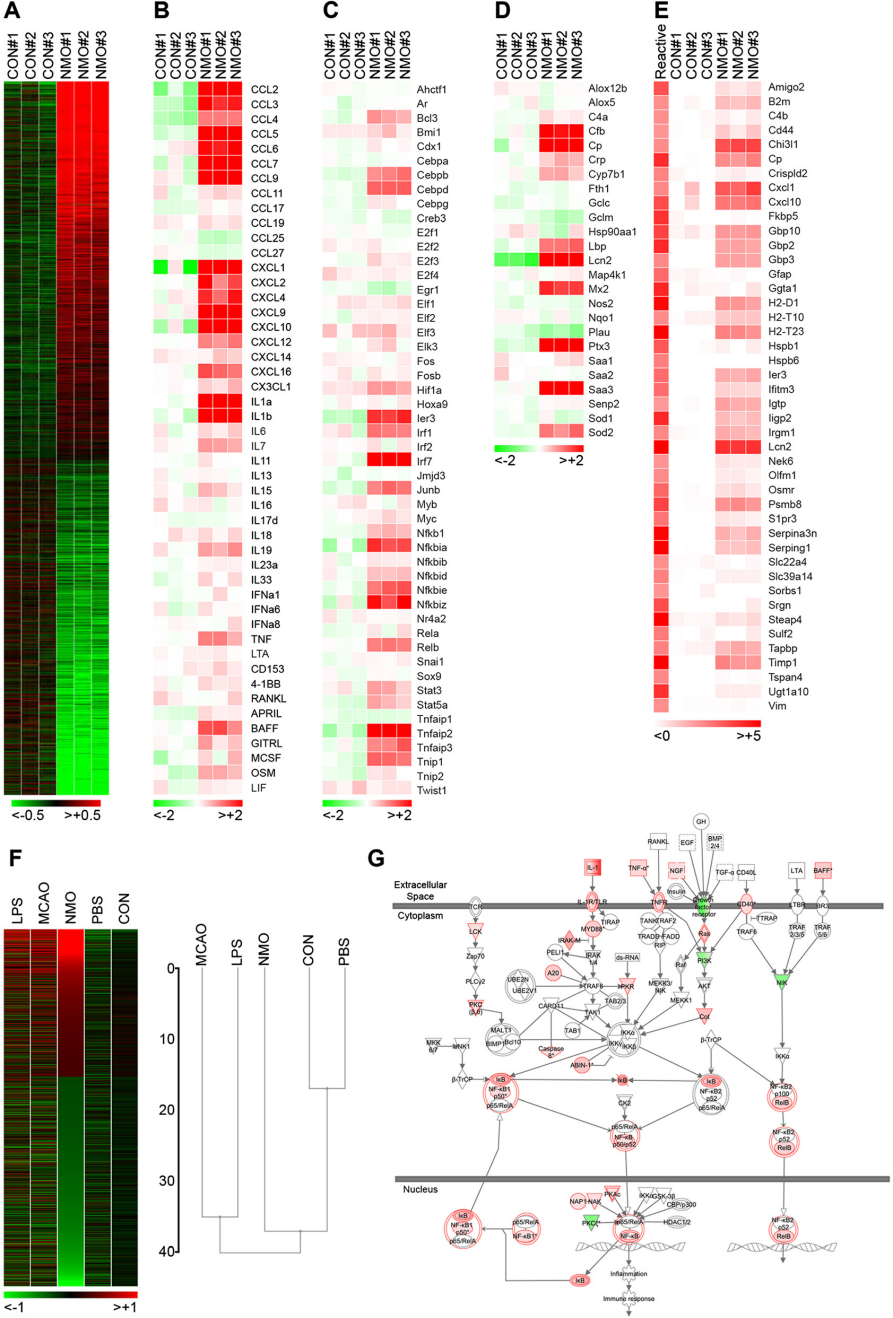
NMO IgG induces expression of inflammatory and reactive astrocyte genes in mouse astroglia. **a**–**f** Gene expression was assessed by microarray analysis of astroglia after 24 h of stimulation with 100 μg/mL NMO IgG (NMO) or control IgG (CON). Changes in expression were calculated by comparison to untreated cultures. **a** A heatmap reveals robust up- and downregulation of numerous genes only in cells stimulated with NMO IgG. Of 22640 genes detected on the microarray, 3628 differed between NMO and CON IgG stimulation at *p* < 0.05. Fold changes for these genes are mapped on a log2 scale, with values downregulated to <−0.5-fold in *green* and values upregulated to >+0.5 shown in *red*. Note that because only significantly changed genes are mapped, there is a discontinuity between the upregulated and downregulated genes. **b** A subset of chemokine and cytokine genes are shown on a log2 scale, with downregulation <−2-fold in *green* and upregulation >+2-fold shown in *red. White* represents zerofold change relative to untreated samples. **c** A subset of genes encoding canonical NFκB-dependent factors are shown on a log2 scale, with downregulation <−2-fold in *green* and upregulation >+2-fold shown in *red*. **d** A subset of NFκB-dependent stress response genes sorted by gene name on log2 scale, with downregulation <−2-fold in *green* and upregulation >+2-fold shown in *red. White* represents zerofold change relative to untreated samples. **e** A published reactive astrocyte transcriptional response pattern (“reactive”) [18] was compared to the changes induced by astroglial stimulation with NMO IgG or CON IgG. These factors were mapped on a log2 scale with <0-fold change shown in *white* and >+5-fold induction shown in *red*. **f** Published data reporting the astrocyte transcriptional response to LPS, middle cerebral artery occlusion (“MCAO”), or PBS [18] were compared to our data for NMO IgG or CON IgG stimulation. The heatmap shows all genes detected on our array; genes with fold change values between −0.26 and +0.26 on a log2 scale following NMO IgG stimulation are excluded from the figure (discontinuity in the NMO lane). Genes downregulated <−2-fold are in *green*, unchanged genes are *black*, and genes upregulated >+2-fold are shown in *red*. A hierarchical cluster analysis showing Euclidean distance and average linkage score was performed in Gitools. The published data used for these comparisons were accessed via the GEO database at NCBI. **g** The NFκB canonical pathway was identified as a top response pathway (*p* = 4.14E-07) using the Ingenuity Pathway Analysis package. Top upstream regulators in this pathway were identified as Stat1 (*z* = 5.530), MyD88 (*z* = 5.603), Ripk2 (*z* = 2.486), and IRF3 (*z* = 2.804). Likewise, IFNγ (*z* = 9.203), IFNβ1 (*z* = 2.412), CSF2 (*z* = 2.789), and TNFα (*z* = 2.121) were identified as top response factors possibly involved in NFκB activation following NMO IgG stimulation. The microarray data were generated in two separate experiments performed with triplicate samples; the purified IgG used for these two experiments were derived from separate patient serum pools (Additional file 1: Table S1). The initial inclusion criteria for detection on the microarray were based on Illumina Beadchip significance calls. Genes exhibiting significant differences between NMO IgG- and CON IgG-stimulated samples were identified using Storey’s positive false-discovery rate for multiple hypothesis testing

The transcriptional response to astroglial stimulation with NMO IgG (NMO) for 24 h was measured using an Illumina mouse WG-6 v2.0 Beadchip (Fig. 2). Compared to cells stimulated with IgG isolated from healthy controls (CON), 3628 genes of the 22,640 genes detected on the array were significantly altered by NMO IgG (Fig. 2a), suggesting a strong transcriptional response to stimulation. A key response included the upregulation of numerous C-C and C-X-C motif chemokine genes, including CCL2, 3, 4, 5, 6, 7, and 9 and CXCL1, 2, 4, 8, 10, 12, and 16 (Fig. 2b). Consistent with our previous findings [7], CCL5 was upregulated 60-fold in astrocytes stimulated with NMO IgG relative to CON IgG. Other significantly upregulated genes of interest were cytokines such as IL-1α, IL-1β, IL-6, and TNFα, suggesting the induction of a broad inflammatory program in astrocytes stimulated with NMO IgG. Genes for several B cell factors, such as B cell activating factor (BAFF), a proliferation inducting ligand (APRIL), and glucocorticoid-induced tumor necrosis factor receptor-related ligand (GITRL) were also upregulated following stimulation with NMO IgG, suggesting a potential interaction between IgG-stimulated astrocytes and localized support for B cell function within the CNS. A large number of canonical NFκB-dependent and NFκB-associated transcription factors (Fig. 2c) and stress response genes (Fig. 2d) were also upregulated in astrocytes stimulated with NMO IgG. Interestingly, the transcript for RELB, the chief transcription factor associated with the alternative NFκB signaling pathway, was strongly upregulated, suggesting that multiple NFκB pathways may be involved in the glial response to NMO IgG over time. Of the stress genes induced by NMO IgG, lipocalin 2 (Lcn2) and ceruloplasmin (Cp) are canonical reactive astrocyte response factors that were strongly upregulated. Indeed, Lcn2 was increased 40-fold in astrocytes stimulated with NMO IgG relative to CON IgG. The induction of a reactive program is further supported by comparison of the NMO IgG-induced response to previously published microarray data from Barres and colleagues [18] characterizing the astrocyte reactome (Fig. 2e). Of note, NMO IgG stimulation induced only a subset of the reactive genes induced by lipopolysaccharide (LPS), suggesting that the NMO-specific response shares some downstream signaling events with LPS-induced reactivity, but does not utilize the same upstream initiators. This conclusion is further supported by comparison of the entire transcriptional response pattern elicited in our system by NMO IgG or CON IgG to the published response induced by LPS, middle cerebral artery occlusion (MCAO), or a phosphate-buffered saline (PBS) control (Fig. 2f) [18]. While NMO IgG stimulation clearly induced a subset of the genes that are also induced by LPS or MCAO, a unique pattern of activation exists in response to the autoantibody. Hierarchical cluster analysis confirmed that LPS and MCAO induced reactive responses that are more closely related to each other than to the NMO IgG-induced response, but that the NMO IgG-induced response is unique from the controls (CON and PBS) (Fig. 2f). Finally, Ingenuity Pathway Analysis [19] revealed that NFκB signaling was a top canonical pathway engaged by stimulation with NMO IgG (Fig. 2g), highlighting the role of this pathway in the observed inflammatory and stress response. We conclude that stimulation of astroglial cultures with NMO IgG induces a distinctive reactive, inflammatory, pro-granulocytic response.

### Stimulation with NMO IgG activates the canonical NFκB signaling pathway in astroglial cultures

**Fig. 3:**
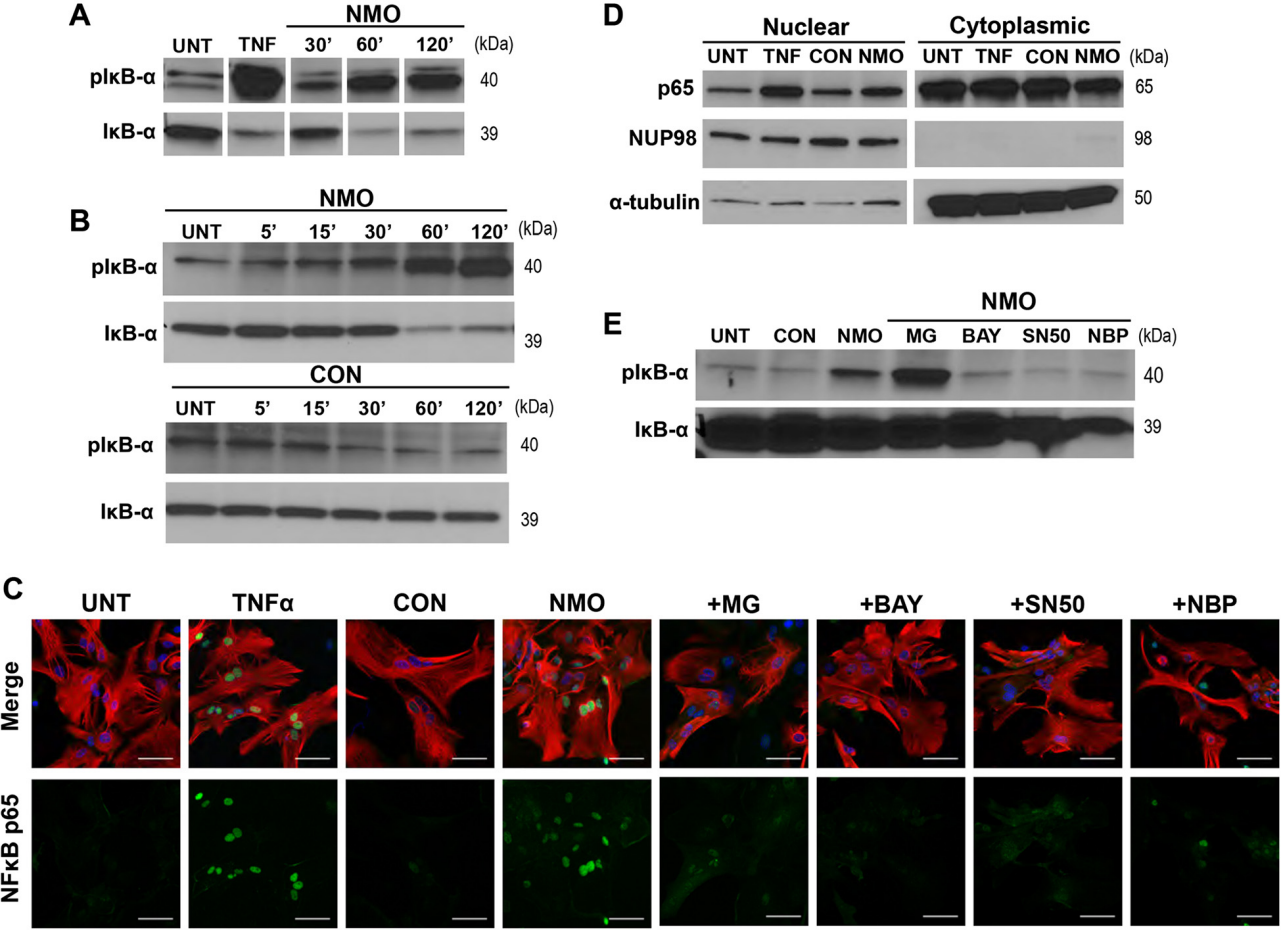
Stimulation with NMO IgG induces canonical NFκB signaling. **a** Lysates (50 μg per lane) from astroglial cells without stimulation (UNT), after 20-min stimulation with 20 ng/mL TNFα (TNF), as a positive control, or after stimulation with 100 μg/mL NMO IgG (NMO) for 30, 60, or 120 min were probed by immunoblot with antibodies specific for either phosphorylated IκB-α (pIκB-α) or total IκB-α. For each antibody, panels are from the same membrane and exposure. **b** Lysates (50 μg per lane) from unstimulated cells (UNT) or following stimulation for 5, 15, 30, 60, or 120 min with 100 μg/mL NMO IgG (NMO) or control IgG (CON) were probed by immunoblot with antibodies for either pIκB-α or IκB-α. Accumulation of pIκB-α protein was evident after stimulation with NMO IgG but not in response to the control IgG. This panel is representative of 2 independent experiments. **c** Cells grown on glass coverslips were probed by immunofluorescence using antibodies against the astrocyte marker glial fibrillary acidic protein (GFAP) (*red*) and NFκB p65 (*green*) after stimulation for 20 min with 20 ng/mL TNFα or stimulation for 60 min with 100 μg/mL NMO IgG (NMO) or CON IgG (CON). In some experiments, NFκB pathway inhibitors were added to the cells for 2 h prior to stimulation. Robust nuclear translocation of p65 was observed following stimulation with NMO IgG and this translocation was blocked when cells were pretreated with NFκB inhibitors. Inhibitor concentrations: 50 μM MG132 (MG), 30 μM BAY 11–7082 (BAY), 30 μM SN50, 30 μM NF-kappa-B essential modulator (NEMO) binding peptide (NBP). These panels are representative of at least 2 independent experiments performed in duplicate. DAPI-labeled nuclei are shown in *blue*. Scale bar: 50 μM. **d** Cellular homogenates were fractionated into nuclear and cytoplasmic components prior to the preparation of lysates. Fractions (10 μg per lane) from unstimulated cells (UNT), cells stimulated for 20 min with 20 ng/mL TNFα (TNF), and cells stimulated for 60 min with 100 μg/mL NMO IgG (NMO) or CON IgG (CON) were probed by immunoblot for levels of p65. The relative purity of the fractions was determined by immunoblotting for the nuclear marker NUP98 and the cytoplasmic marker α-tubulin. Increased nuclear p65 protein was observed following stimulation with NMO IgG as compared to UNT and CON IgG-stimulated samples, consistent with the immunofluorescence in **c**. This blot is representative of 3 independent experiments. **e** Lysates (50 μg per lane) were probed by immunoblot with antibodies for either pIκB-α or total IκB-α following no stimulation (UNT), after 60-min stimulation with 100 μg/mL of CON IgG or NMO IgG, or after stimulation with NMO IgG following pretreatment with NFκB pathway inhibitors. BAY, SN50, and NBP treatments reduced pIκB-α levels following NMO IgG stimulation to the levels observed in unstimulated and CON IgG-stimulated samples. MG132 treatment resulted in accumulation of pIκB-α due to proteasome inhibition, as expected. This blot is representative of at least 2 independent experiments

To characterize astrocytic NFκB signaling in response to stimulation with NMO IgG, we assessed the accumulation of phosphorylated IκB-α by Western blot. Under steady-state conditions, IκB-α is complexed with the p50:p65 (RELA) heterodimer, preventing nuclear translocation and subsequent target gene transcription. Upon exposure to inflammatory signals, IκB-α is phosphorylated by IκB-α kinases (IKK), resulting in IκB-α ubiquitination and consequent targeting to the proteasome. Disassociation of phosphorylated IκB-α (pIκB-α) from the p50:p65 heterodimer exposes a nuclear localization sequence (NLS) that facilitates translocation into the nucleus and concomitant NFκB binding to the promoter region of target genes. We assessed the accumulation of pIκB-α after 20 min of TNFα stimulation as a positive control and after stimulation with NMO IgG for 30, 60, and 120 min (Fig. 3a). pIκB-α accumulation was evident in both TNFα- and NMO IgG-stimulated cells along with a corresponding decrease of total cellular IκB-α, consistent with stimulation-induced degradation. The accumulation of pIκB-α over time was observed as early as 5–15 min after stimulation with NMO IgG and continued to increase over 120 min (Fig. 3b). Critically, exposure to healthy control IgG (CON) under identical conditions did not lead to an increase in IκB-α phosphorylation at any time point (Fig. 3b). Nuclear translocation of NFκB in astrocytes (red) was determined by immunofluorescence, revealing that p65 (green) accumulated in the nucleus (blue) following TNFα stimulation for 20 min and after stimulation with NMO IgG for 60 min (Fig. 3c). NFκB nuclear translocation in response to stimulation with NMO IgG was confirmed by immunoblotting for p65 following fractionation of the cells into nuclear and cytoplasmic constituents (Fig. 3d). The nuclear marker NUP98 and the cytoplasmic marker α-tubulin were used to confirm isolation of a relatively pure nuclear fraction. An increase in nuclear p65 protein was observed following stimulation with either TNFα or NMO IgG, confirming the engagement of NFκB signaling by the autoantibody.

Given the activation of NFκB signaling in astroglial cultures stimulated with NMO IgG, we sought to identify pharmacological inhibitors that would block such signaling and provide insight into the underlying mechanisms of activation. MG132 (MG) is a peptide aldehyde that effectively blocks the proteolytic activity of the 26S proteasome complex, preventing degradation of phosphorylated IκB-α and blocking exposure of the NFκB NLS. BAY 11–7082 (BAY) is an anti-inflammatory compound that inhibits the activity of IKK to prevent phosphorylation of IκB-α. SN50 is an inhibitory peptide that contains the NLS of p50 and blocks nuclear translocation of the active NFκB complex. NFκB essential modulator (NEMO) binding peptide (NBP) is a cell-permeable synthetic peptide corresponding to the NEMO amino-terminal alpha-helical region which blocks the interactions of NEMO with IKK that are critical for activation of the IKK complex. Of note, all inhibitors were tested at a range of concentrations and time points in a standard MTT (3-(4, 5-dimethylthiazol-2-yl)-2,5-diphenyltetrazolium bromide) assay to identify non-toxic concentrations appropriate for treatment (Additional file 5: Figure S2).

Cells were pretreated for 2 h with each inhibitor and then stimulated with NMO IgG for 60 min. As expected, MG132 did not block phosphorylation of IκB-α (Fig. 3e) but did prevent the nuclear translocation of p65 in astrocytes in response to stimulation with NMO IgG (Fig. 3c). The other inhibitors prevented both IκB-α phosphorylation and p65 nuclear translocation (Fig. 3c, e), although NBP was less effective than the other inhibitors at blocking p65 nuclear translocation. The robust inhibition of IκB-α phosphorylation in NBP-treated cells coupled to some evidence of persistent p65 nuclear translocation suggests the parallel activation of an alternative NFκB pathway that is not dependent upon NEMO activation of the IKK complex. In contrast to NBP, the other three inhibitors block elements of both the canonical and alternative NFκB pathways.

### Stimulation with NMO IgG induces NFκB-dependent production and release of pro-granulocytic chemokines

**Fig. 4:**
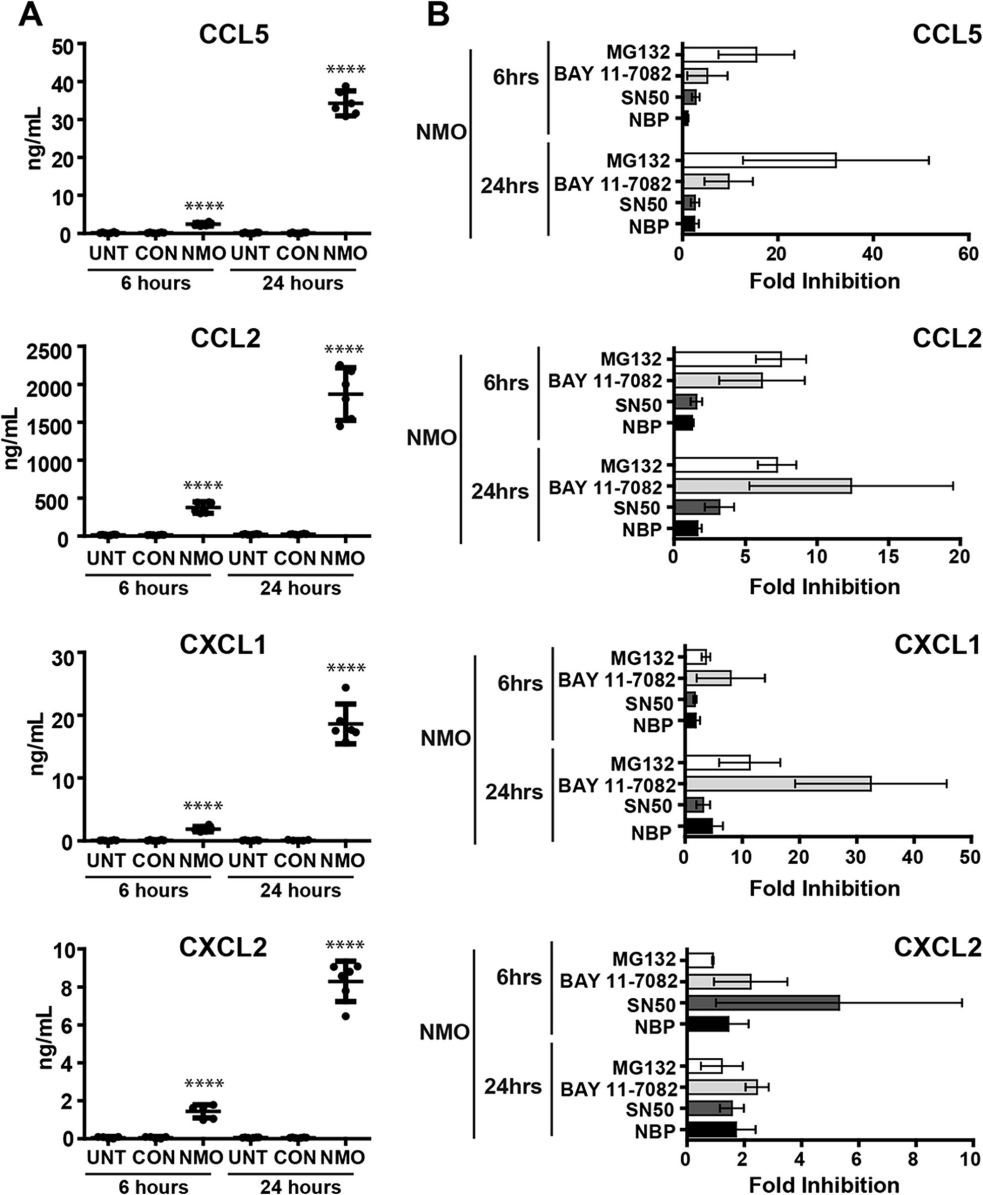
The release of pro-granulocytic chemokines in response to stimulation with NMO IgG is NFκB-dependent. **a** CCL5, CCL2, CXCL1, and CXCL2 release in unstimulated cells (UNT) or following 6- or 24-h stimulation with 100 μg/mL NMO IgG (NMO) or CON IgG (CON) was measured by ELISA. The release of all four chemokines was significantly increased following stimulation with NMO IgG. Stimulation with control IgG did not result in release of any of the chemokines above baseline levels. Data shown are from 2 separate experiments performed in triplicate; each *dot* represents an individual well. The mean response (*solid horizontal bar*) and 95 % confidence intervals (*error bars*) are shown. Significance was determined by one-way analysis of variance (ANOVA) with Bonferroni’s multiple comparison test (****p < 0.0001 versus CON IgG). **b** Fold inhibition of CCL5, CCL2, CXCL1, and CXCL2 release following stimulation with NMO IgG (100 μg/mL) for 6 h or 24 h after pretreatment with NFκB pathway inhibitors was calculated by comparison to cells stimulated with NMO IgG in the absence of inhibitor (amount released with NMO alone/release with NMO+ inhibitor); larger bars equal more robust inhibition. Inhibitor concentrations: 50 μM MG132, 30 μM BAY 11–7082, 30 μM SN50, 30 μM NEMO binding peptide (NBP). MG132 and BAY 11–7082 effectively inhibited CCL5, CCL2, and CXCL1 responses. All of the inhibitors exerted only limited inhibition of CXCL2 release, suggesting that this factor is induced by alternative signaling mechanisms. Data shown are from two separate experiments performed in triplicate. *Error bars* represent the 95 % confidence interval. *p* < 0.001 from three-way ANOVA comparing chemokines, time point, and inhibitors used for treatment

We tested whether astroglial cultures release pro-granulocytic chemokines in response to stimulation with NMO IgG and whether this could be blocked with NFκB inhibitors. We focused on four chemokines: CCL5, a potent chemotactic factor for eosinophils, T cells, and basophils; CCL2, an attractant for monocytes and polymorphonuclear cells; and the neutrophil chemoattractants CXCL1 and CXCL2. Using ELISAs to quantitate chemokine release into supernatants, we found that levels of all four chemokines were significantly higher following 6 or 24 h of stimulation with NMO IgG (NMO) as compared to stimulation with control IgG (CON) or in the absence of stimulation (UNT) (Fig. 4a). Levels of induction after 24 h of stimulation ranged from tens of nanograms per milliliter for CCL5, CXCL1, and CXCL2, to over 2 μg/mL for CCL2. We then measured chemokine release following 6 or 24 h of stimulation with NMO IgG after 2 h of pretreatment with the panel of NFκB inhibitors used above (Fig. 4b). These data are expressed as fold inhibition relative to cells stimulated with NMO IgG in the absence of inhibitor (a larger bar represents greater inhibition). The proteasome inhibitor MG132 blocked more than 95 % of the CCL5 response induced by stimulation with NMO IgG for 24 h and effectively reduced CCL2 and CXCL1 production at levels exceeding tenfold inhibition. The IKK inhibitor BAY 11–7082 was more effective than MG132 at blocking the production of CCL2 and CXCL1, while SN50 and NBP only weakly inhibited production of any of the four factors. Little inhibition of CXCL2 was observed with any of the inhibitors. While SN50 pretreatment induced about 80 % inhibition of CXCL2 production in the 6-h stimulation condition, this effect was overcome by 24 h. The overall lack of inhibition for CXCL2 production mediated by MG132 and BAY 11–7082 suggests that this chemokine is induced by NMO IgG via signaling pathways that largely do not involve NFκB. In contrast, NFκB signaling selectively drives the production of CCL2, CCL5, and CXCL1 following stimulation of astroglia with NMO IgG.

### Bortezomib and PR-957 inhibit NFκB signaling and pro-granulocytic chemokine release in response to stimulation with NMO IgG

**Fig. 5:**
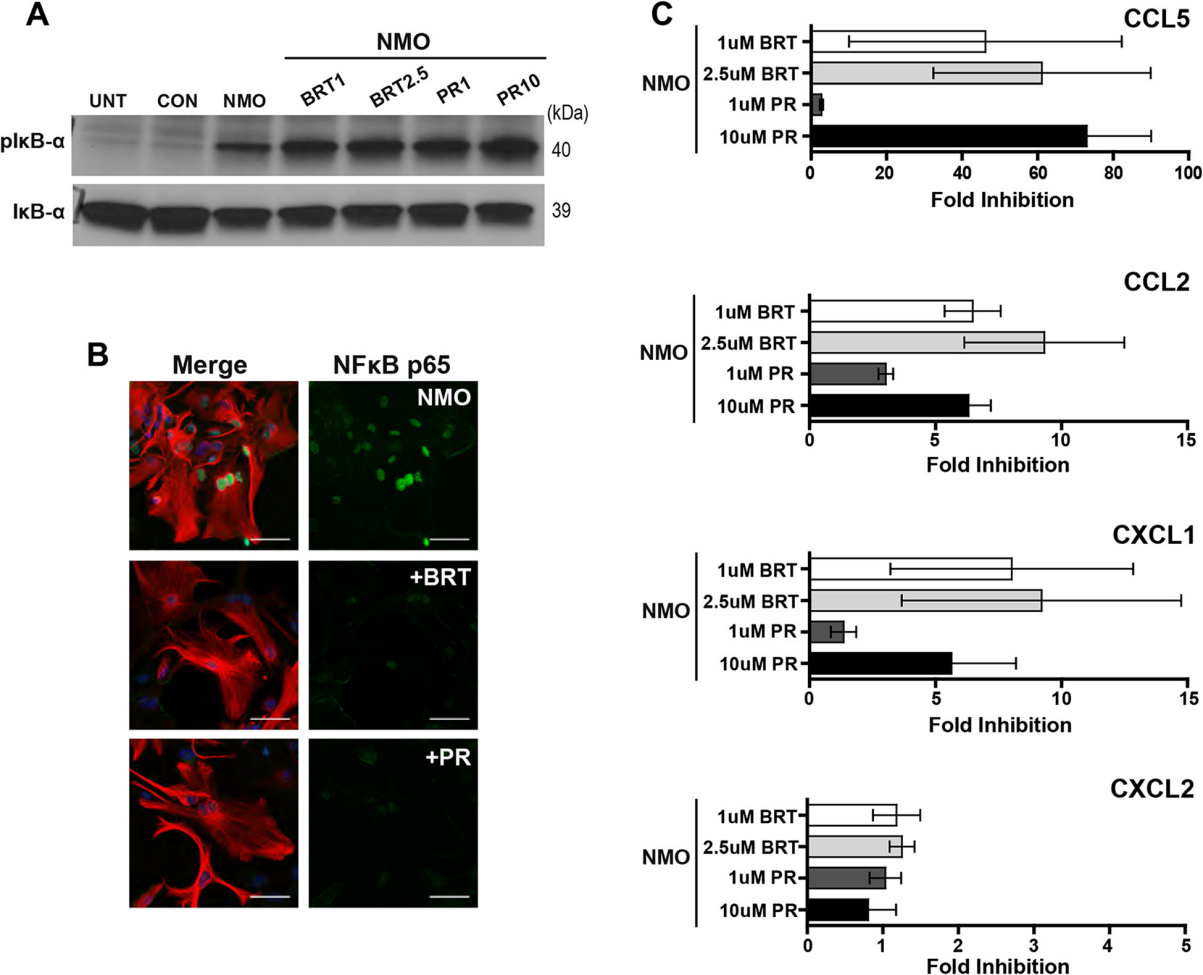
The proteasome inhibitor bortezomib and the immunoproteasome inhibitor PR-957 effectively inhibit NFκB-dependent pro-granulocytic responses induced by NMO IgG. **a** Lysates (30 μg per lane) from unstimulated cells (UNT), following stimulation for 60 min with 100 μg/mL CON IgG (CON) or NMO IgG (NMO), or following stimulation with NMO IgG after pretreatment with bortezomib (BRT 1 μM or 2.5 μM) or PR-957 (PR 1 μM or 10 μM) were probed by immunoblot with antibodies for either pIκB-α or IκB-α. As expected, pIκB-α accumulated in BRT- or PR-treated cells due to impaired proteasome and immunoproteasome function. Blot is representative of 2 independent experiments. **b** Nuclear translocation of NFκB p65 (*green*) in GFAP-labeled cells (*red*) was assessed after stimulation for 60 min with 100 μg/mL NMO IgG alone or following 60-min stimulation with NMO IgG after pretreatment for 2 h with either bortezomib (BRT; 1 μM) or PR-957 (PR; 1 μM). Scale bar: 50 μM. p65 translocation was robustly blocked by both inhibitors. Panel is representative of 2 independent experiments performed in duplicate. **c** The fold inhibition of CCL5, CCL2, CXCL1, and CXCL2 release induced after stimulation of cells for 24 h with 100 μg/mL of NMO IgG in the presence of BRT (1 μM or 2.5 μM) or PR (1 μM or 10 μM) was assessed by ELISA. Both BRT and PR were effective inhibitors of CCL5, CCL2, and CXCL1 responses with little inhibition of CXCL2, as previously observed for MG132. Fold inhibition was calculated as in Fig. 4. Findings are representative of two separate experiments performed in triplicate. *Error bars* show the 95 % confidence interval. *p* < 0.001 from two-way ANOVA comparing chemokines and inhibitors used for treatment

Although MG132, a constitutive proteasome inhibitor, was the least specific NFκB inhibitor that we examined, it showed effective suppression of NMO IgG-induced NFκB signaling and concomitant chemokine release. Considering the successful use of proteasome inhibitors in other diseases, we examined the possible therapeutic relevance of proteasome inhibition in NMO. We tested bortezomib, a dipeptide boronate, approved for treatment of multiple myeloma [20], and the highly selective immunoproteasome inhibitor PR-957, a tripeptide epoxyketone, shown to be efficacious in mouse models of rheumatoid arthritis [21] and SLE [22]. As with MG132, treatment with either bortezomib or PR-957 before stimulation with NMO IgG resulted in the cellular accumulation of pIκB-α (Fig. 5a) due to inhibition of proteasome-mediated degradation. Critically, however, treatment with bortezomib or PR-957 suppressed the NMO IgG-induced nuclear accumulation of NFκB (Fig. 5b) and robustly inhibited the release of CCL2, CCL5, and CXCL1 (Fig. 5c). Indeed, the suppression of CCL5 release by PR-957 approached 99 % inhibition. As with the other NFκB inhibitors, neither bortezomib nor PR-957 inhibited CXCL2 production. These data suggest that inhibition of either the proteasome or immunoproteasome has profound effects on astroglial responses to NMO IgG, leading us to conclude that such inhibition may serve as a therapeutically relevant strategy for suppressing early pathogenic events in NMO.

## Discussion

It is clear that NMO is associated with a unique granulocytic “footprint.” NMO patients often have CSF pleocytosis that includes the presence of polymorphonuclear leukocytes [23–25], an increase in pro-granulocytic chemokines in the CSF [25–28], and notable accumulation of granulocytes in lesions [8, 29, 30] (Fig. 1). In the current conventional model of NMO pathogenesis, terminal complement deposition following binding of NMO IgG to astrocyte endfeet precipitates damage and concomitant granulocyte recruitment into the CNS. This model considers granulocyte recruitment as a downstream *effect* of terminal complement complex formation and tissue injury. However, terminal complement deposition is not a universal feature of all NMO lesions, and therapeutics targeting complement inhibition are efficacious in only some patients [31], suggesting that complement-mediated tissue destruction in NMO lesions may represent only one possible pathogenic mechanism. We contend that there are also early, sub-lytic, and highly inflammatory astrocytic responses to NMO IgG that contribute to early granulocytic recruitment. In our model, granulocyte recruitment is an upstream *cause* of injury and is triggered by astrocyte signaling, rather than astrocyte death. We observed clear granulocytic accumulation in NMO patient tissue that was not dependent on complement deposition, active demyelination, or tissue destruction, along with evidence of reactive astrocytes in these regions (Fig. 1). Building on these observations, we utilized an astroglial culture system to examine the rapid cellular and molecular events induced by stimulation with NMO IgG in the absence of exogenous complement. We found that such stimulation engaged a highly inflammatory and reactive astrocyte transcriptional program that included the upregulation of numerous genes encoding pro-granulocytic chemokines (Fig. 2). Further analysis of the transcriptional program initiated by stimulation with NMO IgG revealed that the NFκB signaling pathway was significantly upregulated. Confirming engagement of the NFκB signaling pathway, we observed phosphorylation of IκB-α and nuclear translocation of the NFκB transcription factor p65 in astrocytes following stimulation with NMO IgG. Treatment with a spectrum of NFκB inhibitors effectively blocked these responses (Fig. 3). Finally, we found that the potent pro-granulocytic chemokines CCL5, CCL2, CXCL1, and CXCL2 were released by cells following stimulation with NMO IgG, and that the release of all except CXCL2 was effectively blocked by inhibition of NFκB (Fig. 4), including inhibition using clinically relevant proteasome inhibitors (Fig. 5).

NFκB is a well-studied master regulator of autoimmunity that is crucial for both inflammation and immune tolerance. While NFκB activation occurs transiently in the course of a normal immune response, chronic activation of this signaling pathway in target tissues is associated with pathogenesis in many autoimmune diseases [32]. Importantly, therapeutic targeting of the NFκB signaling pathway is clinically feasible and may provide a strategy for controlling the transition from normal immunity to autoimmunity. In our model, proteasome inhibition by MG132, bortezomib, and PR-957 effectively blocked the release of several pro-granulocytic chemokines (Figs. 3, 4, and 5). The efficacy of bortezomib is of interest due to its current therapeutic use in multiple animal models and in patients. Bortezomib treatment results in decreased inflammation in animal models of contact hypersensitivity [33], allograft rejection [34], and SLE [35]. Bortezomib is an approved therapy for multiple myeloma patients where treatment inhibits NFκB and induces myeloma cell apoptosis [36]. Bortezomib was also recently shown to halt autoantibody production and kill plasma cells in a murine model of myasthenia gravis [37]. Of note, long-lived plasma cells, due to the metabolic demand required by a high rate of IgG production, are particularly sensitive to proteasome inhibition [35] but are generally resistant to immunosuppressive drugs, including anti-CD20 antibodies such as rituximab that are currently used to functionally inhibit or deplete B cell populations [38]. Considering the upregulation of B cell-related factors and other inflammatory drivers in astrocytes stimulated with NMO IgG (Fig. 2) and the published evidence for intrathecal production of IgG in NMO patients [39, 40], therapeutic proteasome inhibition may provide the dual advantage of simultaneously targeting both the inflammatory astrocytic response and plasma cell survival—essentially blocking both the stimulator and the concomitant stimulation. Furthermore, the clinical efficacy of bortezomib coupled with immunomodulators such as lenalidomide or methylprednisolone in transplant and multiple myeloma patients suggests that combined drug strategies may confer significant therapeutic advantage in complex inflammatory diseases such as NMO.

One complication with bortezomib is the potential for peripheral neuropathy at therapeutic doses. Therefore, we expanded our study to include testing of PR-957 (ONX-914), an immunoproteasome inhibitor that has shown efficacy in an animal model of rheumatoid arthritis (RA) at far lower doses than are employed for the proteasome inhibitor carfilzomib [21]. Similar results were found in mouse models of colitis, MS [41], and SLE [22], where PR-957 treatment significantly inhibited the production of pro-inflammatory cytokines and reduced the severity of disease symptoms. The immunoproteasome is mostly found in cells of the immune system but can be expressed in other cells, including astrocytes, upon exposure to various stressors [42]. In a pro-inflammatory environment, virtually all newly synthesized 20S proteasomes incorporate inducible subunits associated with the immunoproteasome rather than constitutive subunits [43]. Treatment with PR-957 in our system resulted in efficient blockade of NFκB p65 translocation in astrocytes and inhibited the release of CCL5, CCL2, and CXCL1 (Fig. 5). These results suggest that astrocytic inflammatory responses in patients with NMO may be targeted by specific inhibition of the immunoproteasome. Finally, evidence that immunoproteasome inhibition also suppresses the production of autoantibodies [22] and selectively targets plasma cell survival [21] further suggests that such a strategy may confer significant disease-modifying effects in patients with NMO. We propose that the use of drugs such as PR-957 in NMO patients may reduce or block disease pathogenesis via parallel pathways that reduce astrocyte reactivity, suppress CNS inflammation, block granulocyte recruitment, reduce production of NMO IgG, and deplete autoantibody-producing plasma cells that are resistant to current therapies such as rituximab [38].

One limitation of our study is the absence of a suitable animal model in which to test our hypothesis. Existing mouse models of NMO rely on the concurrent initiation of active (via immunization) or passive (via transfer of myelin-specific T cells) EAE [44–46], co-injection of NMO IgG and human complement factors into the CNS [47–49], or injection of the proinflammatory cytokine IL-1β directly into the brain to drive granulocyte recruitment and complement factor production [50]. The “success” of these models is due to inflammatory modulation of the blood–brain barrier and direct induction of complement-mediated pathology. Unfortunately, the use of any of these models would compromise the analysis of early astrocytic responses to NMO IgG and are therefore not suitable to test our hypothesis. Another area in which a suitable animal model is necessary but currently unavailable is the analysis of antibody-induced granulocytic recruitment in the absence of overt tissue destruction or complement deposition. A pathogenic role for neutrophils in CNS autoimmune disease was suggested by the occurrence of severe exacerbations in some MS and NMO patients given recombinant granulocyte-colony stimulating factor (G-CSF) which stimulates the function and proliferation of granulocytes [51]. Although the presence of neutrophils in typical MS lesions is rare, studies in the EAE model indicate that neutrophils are found at high frequency in the CNS parenchyma during the preclinical phase, increase dramatically in the meninges both preclinically and at relapse, and may potentiate the formation of lesions by mediating the breakdown of the blood–brain or blood-cerebrospinal fluid barriers or by stimulating the maturation of local antigen-presenting cells [52]. With regard to eosinophils, degranulated cells are found in both NMO meninges and early lesions [30]. While historically recognized as endstage effectors in parasitic immunity and allergic diseases, it is quite likely that eosinophils directly contribute to tissue injury in NMO via release of cytokines, chemokines, lipid mediators, oxygen burst components, and cytotoxic granule cationic proteins [53]. Elucidating and targeting the mechanisms that recruit granulocytes to early NMO lesions and discovering strategies to inhibit the initial damage triggered by these cells will require the creation of animal models that do not involve the tautological induction of complement-dependent injury or the initiation of inflammation that does not build from a reactive astrocyte response.

## Conclusions

The current study provides evidence for an expanded model of NMO pathogenesis and supports the possible therapeutic use of proteasome and immunoproteasome inhibitors in patients with NMO. Although terminal complement deposition-driven granulocyte recruitment and lytic tissue destruction are definite components of NMO pathogenesis, considerable clinical and histopathological evidence indicates that NMO lesions are potentially reversible [8, 54]. Our findings suggest that early events in disease pathogenesis may involve NMO IgG-induced NFκB-dependent signaling in astrocytes that results in the creation of a pro-granulocytic inflammatory milieu, leading to lesion development that is multimodal and amenable to therapeutic intervention at several points. While complement inhibition will prevent the most tissue destructive aspects of NMO lesion development, upstream interventions that impact the reactive and inflammatory astrocyte response, reduce granulocyte infiltration, and short-circuit the functionally disruptive genesis of acute lesions are needed to reverse symptoms and prevent irreversible damage in NMO patients.

## Additional file

Additional file 1:
**Table S1.** NMO serum pools used in this study. (PDF 28.4 kb) Additional file 2: Table S2. Representative NMO serum pool. NMO patient sera were pooled from 5 males and 36 females ranging in age from 14 to 79, with a median age of 48. (PDF 37.4 kb) Additional file 3: Table S3. Control sera were pooled from age- and sex-matched donors. (PDF 37.9 kb) Additional file 4: Figure S1. Astrocyte responses are independent of NMO serum pool provenence. Historical results generated using multiple different pools of NMO patient serum indicate that the NFκB response and chemokine production triggered by NMO IgG stimulation are not restricted to a specific NMO patient pool. Astrocytes were stimulated for 60 min with 100 µg/mL purified CON IgG (A) or NMO IgG (B) from serum pools prepared in 2012 (see Table S1). Only the NMO IgG induced nuclear localization of NFκB. Green = NFκB; red = GFAP. (C) Comparison of CCL5 release from astrocytes, as assessed by ELISA, following stimulation with pooled serum prepared in 2013 (see Table S1) or IgG purified from these pools. Only NMO serum and purified NMO IgG induced CCL5 production above untreated (UNT) levels. (D) The impact of proteasome inhibition (MG-132) and NFκB inhibition (BAY 11-7082) on CCL2 production in response to stimulation with NMO IgG purified from a 2014 NMO serum pool (see Table S1). (E) Evidence of IκB phosphorylation at 45 and 60 min after treatment with NMO IgG purified from a 2012 NMO patient serum pool (see Table S1). Control IgG (CON) has no impact on IκB phosphorylation. By way of comparison, the majority of figures shown in the main manuscript are representative of treatments with serum pools prepared in 2014 and 2015. (PDF 276 kb) Additional file 5: Figure S2. Assessment of cell viability during NFκB and proteasome inhibitor treatment. A standard colorimetric MTT assay was used to assess cell viability following treatment with different concentrations of the NFκB inhibitors employed in our study. (A) Cells were exposed to high concentrations (10, 50, 100 µM) of MG132, BAY 11-7082, NBP, and SN50 for 24 hours. (B) Cell viability was determined using an extended concentration curve (shown in µM) and multiple time points (2, 6, 24 hours) for BAY 11-7082, NBP, SN50, bortezomib and PR-957. Data are representative of 1-2 separate experiments performed in duplicate or triplicate. Each dot represents an individual well. Percent viability was calculated as (sample absorbance/untreated absorbance)*100. (PDF 116 kb)
