# Supplementary material for: NFκB signaling drives pro-granulocytic astroglial responses to neuromyelitis optica patient IgG
Source: J Neuroinflammation. 2015 Sep 30;12:185. doi: 10.1186/s12974-015-0403-8 (PMC4590277; doi:10.1186/s12974-015-0403-8)
Supplement: Additional file 1: — Table S1. NMO serum pools used in this study. (PDF 28.4 kb) [file 12974_2015_403_MOESM1_ESM.pdf]

Table S1: NMO serum pools used in this study.

**S1. Study pools: NMO**				
**year**	**# female patients**	**# male patients**	**age range**	**IgG yield**
2011	15	0	32-77	#1 @ 15.5 mg/mL
2012	20	5	9-69	#1 @ 18.8 mg/mL<br>#2 @ 22 mg/mL
2013	20	3	14-72	#1 @ 56 mg/mL (conc)<br>#2 @ 20.7 mg/mL
2014	36	5	14-79	#1 @ 12.7 mg/mL<br>#2 @ 14.3 mg/mL
2015	38	7	19-75	#1 @ 19.4 mg/mL<br>#2 @ 23.2 mg/mL
