# Supplementary material for: NFκB signaling drives pro-granulocytic astroglial responses to neuromyelitis optica patient IgG
Source: J Neuroinflammation. 2015 Sep 30;12:185. doi: 10.1186/s12974-015-0403-8 (PMC4590277; doi:10.1186/s12974-015-0403-8)
Supplement: Additional file 2: Table S2. — Representative NMO serum pool. NMO patient sera were pooled from 5 males and 36 females ranging in age from 14 to 79, with a median age of 48. (PDF 37.4 kb) [file 12974_2015_403_MOESM2_ESM.pdf]

**Table S2**: Representative NMO serum pool. NMO patient sera were pooled from 5 males and 36 females ranging in age from 14 to 79, with a median age of 48.

| S2. NMO patient cohort for serum collection and NMO IgG isolation (2014 pool) | | | |
|---|---|---|---|
| **Sample ID** | **Sex** | **DOB** | **Age** |
| 082 | M | 12/31/1953 | 59 |
| 083 | F | 5/15/1993 | 20 |
| 084 | F | 8/20/1943 | 69 |
| 085 | F | 10/27/1956 | 56 |
| 086 | F | 2/18/1942 | 71 |
| 087 | F | 12/11/1992 | 20 |
| 088 | F | 11/18/1941 | 71 |
| 089 | M | 6/13/1946 | 67 |
| 090 | F | 1/24/1968 | 45 |
| 091 | F | 6/26/1943 | 70 |
| 092 | F | 2/4/1999 | 14 |
| 093 | F | 9/27/1955 | 57 |
| 094 | M | 3/2/1978 | 35 |
| 095 | F | 9/20/1963 | 49 |
| 096 | F | 7/26/1969 | 44 |
| 097 | F | 5/15/1993 | 20 |
| 098 | F | 8/24/1985 | 27 |
| 099 | F | 9/20/1963 | 49 |
| 100 | F | 1/1/1941 | 72 |
| 101 | F | 6/17/1988 | 25 |
| 102 | F | 6/18/1963 | 50 |
| 103 | F | 1/3/1965 | 48 |
| 104 | F | 2/18/1942 | 71 |
| 105 | F | 5/3/1979 | 35 |
| 106 | M | 6/6/1958 | 55 |
| 107 | F | 9/11/1984 | 29 |
| 108 | F | 3/8/1989 | 25 |
| 109 | F | 7/12/1951 | 62 |
| 110 | F | 12/20/1983 | 30 |
| 111 | F | 9/20/1966 | 47 |
| 112 | F | 10/26/1972 | 41 |
| 113 | F | 6/14/1972 | 41 |
| 114 | F | 12/21/1962 | 51 |
| 115 | F | 2/6/1986 | 28 |
| 116 | F | 4/24/1971 | 43 |
| 117 | F | 2/29/1988 | 26 |
| 118 | F | 2/13/1941 | 73 |
| 119 | F | 10/17/1933 | 79 |
| 120 | F | 10/24/1943 | 70 |
| 121 | M | 12/7/1994 | 19 |
| 122 | F | 9/24/1965 | 48 |
