# Supplementary material for: NFκB signaling drives pro-granulocytic astroglial responses to neuromyelitis optica patient IgG
Source: J Neuroinflammation. 2015 Sep 30;12:185. doi: 10.1186/s12974-015-0403-8 (PMC4590277; doi:10.1186/s12974-015-0403-8)
Supplement: Additional file 3: Table S3. — Control sera were pooled from age- and sex-matched donors. (PDF 37.9 kb) [file 12974_2015_403_MOESM3_ESM.pdf]

Table S3: Control sera were pooled from age- and sex-matched donors.

**S3. CON patient cohort for serum collection and CON IgG isolation**			
**Sample ID**	**Sex**	**DOB**	**Age**
058	M	12/31/1953	59
059	F	5/15/1993	20
060	F	8/20/1943	69
061	F	10/27/1956	56
062	F	2/18/1942	71
063	F	12/11/1992	20
064	F	11/18/1941	71
065	M	6/13/1946	67
066	F	1/24/1968	45
067	F	6/26/1943	70
068	F	2/20/1961	52
069	F	2/4/1999	14
070	F	9/27/1955	57
071	M	3/2/1978	35
072	F	9/20/1963	49
073	F	7/26/1969	44
074	F	5/15/1993	20
075	F	8/24/1985	27
076	F	9/20/1963	49
077	F	1/1/1941	72
078	F	6/17/1988	25
079	F	6/18/1963	50
080	F	1/3/1965	48
081	F	2/18/1942	71
058	M	12/31/1953	59
059	F	5/15/1993	20
060	F	8/20/1943	69
061	F	10/27/1956	56
062	F	2/18/1942	71
063	F	12/11/1992	20
064	F	11/18/1941	71
065	M	6/13/1946	67
066	F	1/24/1968	45
067	F	6/26/1943	70
068	F	2/20/1961	52
069	F	2/4/1999	14
070	F	9/27/1955	57
071	M	3/2/1978	35
072	F	9/20/1963	49
073	F	7/26/1969	44
074	F	5/15/1993	20
