# Supplementary material for: NFκB signaling drives pro-granulocytic astroglial responses to neuromyelitis optica patient IgG
Source: J Neuroinflammation. 2015 Sep 30;12:185. doi: 10.1186/s12974-015-0403-8 (PMC4590277; doi:10.1186/s12974-015-0403-8)
Supplement: Additional file 4: Figure S1. — Astrocyte responses are independent of NMO serum pool provenence. Historical results generated using multiple different pools of NMO patient serum indicate that the NFκB response and chemokine production triggered by NMO IgG stimulation are not restricted to a specific NMO patient pool. Astrocytes were stimulated for 60 min with 100 µg/mL purified CON IgG (A) or NMO IgG (B) from serum pools prepared in 2012 (see Table S1). Only the NMO IgG induced nuclear localization of NFκB. Green = NFκB; red = GFAP. (C) Comparison of CCL5 release from astrocytes, as assessed by ELISA, following stimulation with pooled serum prepared in 2013 (see Table S1) or IgG purified from these pools. Only NMO serum and purified NMO IgG induced CCL5 production above untreated (UNT) levels. (D) The impact of proteasome inhibition (MG-132) and NFκB inhibition (BAY 11-7082) on CCL2 production in response to stimulation with NMO IgG purified from a 2014 NMO serum pool (see Table S1). (E) Evidence of IκB phosphorylation at 45 and 60 min after treatment with NMO IgG purified from a 2012 NMO patient serum pool (see Table S1). Control IgG (CON) has no impact on IκB phosphorylation. By way of comparison, the majority of figures shown in the main manuscript are representative of treatments with serum pools prepared in 2014 and 2015. (PDF 276 kb) [file 12974_2015_403_MOESM4_ESM.pdf]

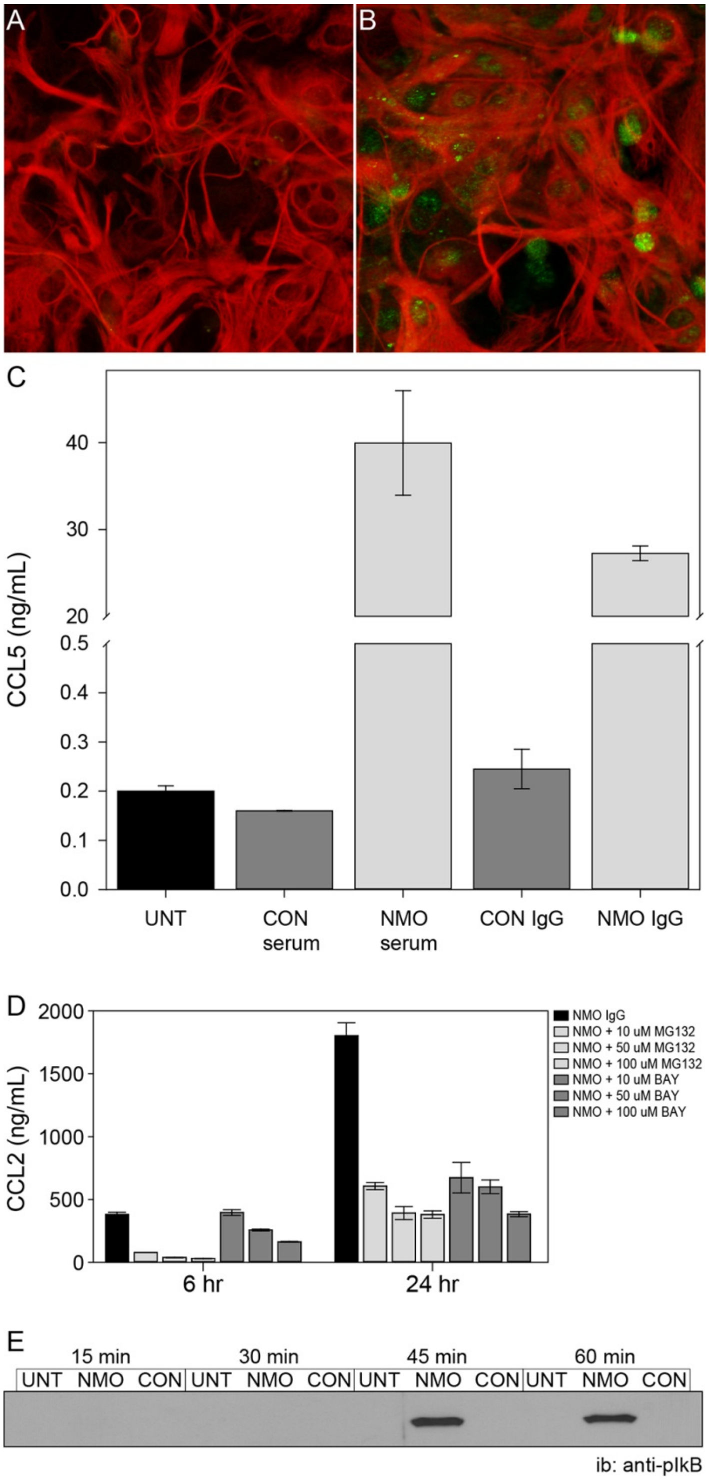

Supplementary Figure 1. Astrocyte responses are independent of NMO serum pool provenence. Historical results generated using multiple different pools of NMO patient serum indicate that the NFκB response and chemokine production triggered by NMO IgG stimulation are not restricted to a specific NMO patient pool. Astrocytes were stimulated for 60 min with 100

µg/mL purified CON IgG (A) or NMO IgG (B) from serum pools prepared in 2012 (see Table S1). Only the NMO IgG induced nuclear localization of NFκB. Green = NFκB; red = GFAP. (C) Comparison of CCL5 release from astrocytes, as assessed by ELISA, following stimulation with pooled serum prepared in 2013 (see Table S1) or IgG purified from these pools. Only NMO serum and purified NMO IgG induced CCL5 production above untreated (UNT) levels. (D) The impact of proteasome inhibition (MG-132) and NFκB inhibition (BAY 11-7082) on CCL2 production in response to stimulation with NMO IgG purified from a 2014 NMO serum pool (see Table S1). (E) Evidence of IκB phosphorylation at 45 and 60 min after treatment with NMO IgG purified from a 2012 NMO patient serum pool (see Table S1). Control IgG (CON) has no impact on IκB phosphorylation. By way of comparison, the majority of figures shown in the main manuscript are representative of treatments with serum pools prepared in 2014 and 2015.
