# Supplementary material for: NFκB signaling drives pro-granulocytic astroglial responses to neuromyelitis optica patient IgG
Source: J Neuroinflammation. 2015 Sep 30;12:185. doi: 10.1186/s12974-015-0403-8 (PMC4590277; doi:10.1186/s12974-015-0403-8)
Supplement: Additional file 5: Figure S2. — Assessment of cell viability during NFκB and proteasome inhibitor treatment. A standard colorimetric MTT assay was used to assess cell viability following treatment with different concentrations of the NFκB inhibitors employed in our study. (A) Cells were exposed to high concentrations (10, 50, 100 µM) of MG132, BAY 11-7082, NBP, and SN50 for 24 hours. (B) Cell viability was determined using an extended concentration curve (shown in µM) and multiple time points (2, 6, 24 hours) for BAY 11-7082, NBP, SN50, bortezomib and PR-957. Data are representative of 1-2 separate experiments performed in duplicate or triplicate. Each dot represents an individual well. Percent viability was calculated as (sample absorbance/untreated absorbance)*100. (PDF 116 kb) [file 12974_2015_403_MOESM5_ESM.pdf]

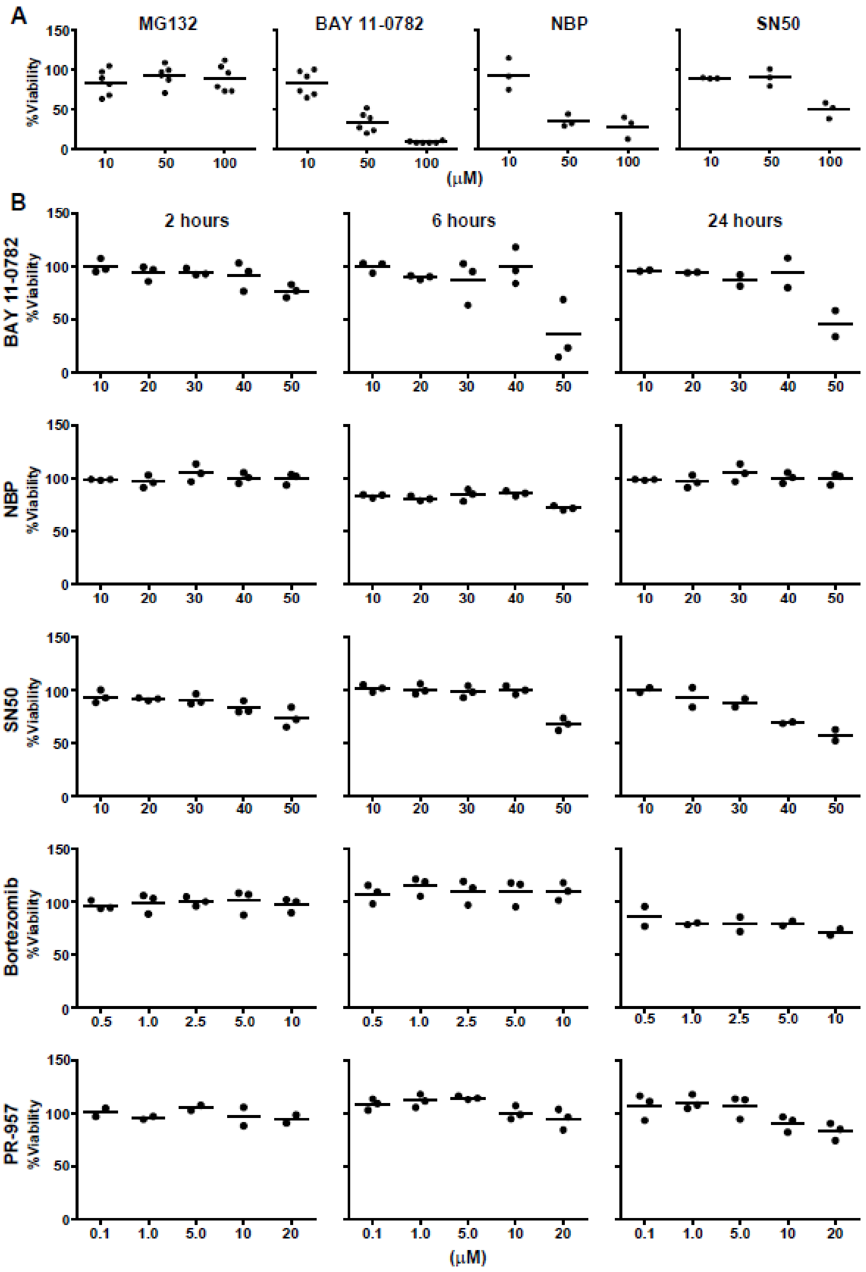

Supplementary Figure 2. Assessment of cell viability during NFκB and proteasome inhibitor treatment. A standard colorimetric MTT assay was used to assess cell viability following treatment with different concentrations of the NFκB inhibitors employed in our study. (A) Cells were exposed to high concentrations (10, 50, 100 μM) of MG132, BAY 11-7082, NBP, and SN50 for 24 hours. (B) Cell viability was determined using an extended concentration curve (shown in μM) and multiple time points (2, 6, 24 hours) for BAY 11-7082, NBP, SN50, bortezomib and PR-957. Data are representative of 1-2 separate experiments performed in duplicate or triplicate. Each dot represents an individual well. Percent viability was calculated as (sample absorbance/untreated absorbance)*100.
